# Commentary: Is There a Lack of Insight on the Anatomy Teaching Adaptations Made by “Cadaver-Free” Medical Schools in Response to the COVID-19 Pandemic?

**DOI:** 10.1007/s40670-023-01822-y

**Published:** 2023-07-01

**Authors:** Eleni Patera

**Affiliations:** Section of Anatomy, St George’s University of London, London, UK

**Keywords:** Dissection, Anatomy education, Pre-clinical medical education

## Abstract

The COVID-19 pandemic negatively impacted anatomy education as it ceased face-to-face anatomy teaching sessions and laboratory practicals. In the past 2 years, a growing body of literature has been dedicated to the adaptations made in the teaching of anatomy predominantly by medical schools who employ cadaveric dissection and prosection-based practicals to teach anatomy. Despite this, there is dearth of evidence in terms of the challenges that medical schools who do not use cadaveric dissection or prosected specimens to teach anatomy faced as well as the adaptations they made in response to the pandemic.

Anatomy as a subject has been long characterized as a visual subject, and there is no doubt that it is a non-theoretical subject that requires students to have an adequate exposure to the subject by acquiring hands on experience via cadaveric or prosectorium-based laboratory practicals, patient simulations, medical imaging training, and other teaching practices that aim to increase students’ spatial ability and enhance their visualization over anatomical structures [1]. The COVID-19 pandemic has negatively impacted higher education as it imposed unforeseen challenges to university stakeholders [2]. Medical schools needed to shut down to keep students safe, simultaneously ensuring teaching and learning continuity amid the pandemic. This has been particularly difficult for faculty members who teach anatomy and students who have an anatomy component in their curriculum. On one hand, anatomy educators needed to ensure that their teaching material was delivered completely online, that their online teaching content and learning resources were accessible to all students, and that student assessment was planned and implemented according to the new online teaching setting [3, 4]. On the other hand, this transition has been difficult for students as well, as their face-to-face learning environment has been suddenly shifted to an online virtual environment who required them to attend their lectures virtually by sitting in front of their computer for long hours. It is possible that undergraduate pre-clinical medical students find the transition from secondary education to tertiary education strenuous [5]. Therefore, it can be assumed that the rapid shift to online teaching during the pandemic has further stretched their process of adaptation.

Currently, in the literature, there is a substantial number of articles dedicated to the employment of cadaveric dissection or prosected specimens in medical schools for the teaching of anatomy [6–8]. However, it seems like this teaching method is controversial as it has anatomy educators’ opinion divided into those who believe that exposing medical students to cadaveric dissection or prosected specimens is indispensable and anatomy educators who believe that the teaching of anatomy can take place by other means such as physical 3D models as well as digital resources [9–11]. While this debate is still ongoing, several educational concerns regarding the teaching and learning of anatomy are a common concern for anatomy and medical educators globally irrespective of the anatomy teaching methods that are employed at each medical institution.

In the past 2 years, a large body of literature has addressed the challenges that the COVID-19 pandemic imposed on medical schools as well as the adaptations that have been made in response to it [12–15]. Since the COVID-19 pandemic ceased face-to-face anatomy teaching sessions, there has been a growing body of literature dedicated to the adaptations made in the teaching of anatomy predominantly by medical schools who employ cadaveric dissection and prosection-based practicals for the teaching of anatomy [3, 16–18]. Remarkably, the field of anatomy education has been prolongedly affected by various challenges prior the advent of the COVID-19 pandemic. Such challenges encompass the substantial reduction in the anatomy teaching hours, the possibility of medical curricula being overloaded, the lack of trained staff to teach anatomy as well as the high cost for obtaining or maintaining specific educational resources [19]. Some of these pre-existing challenges have been exacerbated by the pandemic. For instance, obtaining human bodies via body donation for cadaveric or prosection-based teaching has been a difficult task for many reasons. Some of these include cadavers being in short supply as well as the high cost for establishing a cadaver lab and the fees for transporting, storing, and embalming the cadavers [19–21]. Furthermore, the pandemic has made the acquisition of human bodies for teaching purposes much more difficult as it was necessary to ensure that the donated bodies a medical institution receives are safe to be used [21]. Despite this, even if the acquisition of donated bodies during the pandemic was feasible, the conduction of cadaveric or prosectorium-based labs amidst the pandemic might not have been feasible due to the health and safety measures that have been taken in response to minimize the spread of the disease. Furthermore, there are specific medical schools where the number of pre-clinical medical students exceeds the 400–500. In addition, students from health allied professions often receive dissection or prosection based laboratory practicals; hence, adapting the anatomy laboratory practicals by reducing the number of students attending each laboratory practical might still not have been feasible due to time restrictions and a potential shortage of anatomy teaching staff members. Recent evidence from the literature reports the adaptations that medical schools who employ cadaveric dissection and/or prosectorium-based practicals for the teaching of anatomy made in response to the anatomy laboratory practicals being ceased due to the pandemic. An adaptation made at the University of Glasgow for undergraduate anatomy students was to simulate cadaveric dissection by using virtual resources such as cadaveric dissection videos and a 3D real-time virtual anatomy atlas [22]. Yoo et al. [16] reported that at the Korean University College of Medicine in Korea, anatomy educators provided students with pre-recorded laboratory dissection videos and access to a 3D program that explained various anatomical structures. The medical school at Anglia Ruskin University ceased the anatomy laboratory practicals; therefore, to overcome this, staff have signposted students to appropriate online resources including YouTube videos [23]. A research study by Shin et al. [18] evaluated anatomy education in the USA before and during the pandemic. The authors reported that prior the pandemic, most medical schools relied on cadaveric dissection as an interactive method to allow their students to gain hands on experience with the subject. Subsequently, the authors reported that the in-person anatomy laboratory practicals have been replaced by online images of cadaveric dissection that has been previously performed by experienced anatomists [18]. In a research article, Attardi and co-authors [3] document the changes in gross anatomy education before and during the COVID-19 pandemic. The authors report that during the pandemic, gross anatomy laboratory teaching in medical schools in the USA took place either synchronously, asynchronously, or in a mixed format [3]. All three types of laboratory delivery modalities took place either in-person or not in-person or a combination of both in the case of the mixed laboratory delivery modality [3].

Another major aspect of the anatomy teaching that needed to be amended due to the pandemic was the delivery of in-person didactic lectures. Some of the adaptations that were made in response to the pandemic were shifting the in-person didactic lectures to virtual live lecture sessions that were either recorded or not or pre-recorded lectures [18]. Another adaptation that has been reported in the literature was the employment of the flipped classroom approach as well as pre-readings [18]. Student assessment has been another arduous challenge during the COVID-19 era, as anatomy and medical educators needed to re-designed student assessment so that it is analogous to the new online teaching setting and fair in terms of how students will be assessed based on how they have been taught. Educators needed to explore which online assessment methods can support students’ learning as according to Gibbs and Simpson [24], assessment has a great influence on students’ motivation to study and to what extent.

It is possible that the adaptations that were made in terms of the delivery of didactic lectures and methods for assessing students’ knowledge have been very similar if not the same across medical schools that employ cadaveric dissection and prosection-based laboratory practicals and medical schools that do not. Nevertheless, a couple of key questions worth to be addressed are the following: “To what extent have “cadaver-free” medical schools been affected during the pandemic?” and “What anatomy teaching adaptations did these medical schools make in response to the pandemic?”. It can be assumed that prior the pandemic, medical schools who do not employ cadaveric dissection or prosection-based practicals perhaps run small group teaching sessions were students have access to anatomy physical models such as bones and plastic anatomical model sets such as human torso models. Additionally, it is already known from previous published literature prior the pandemic that medical schools who do not use cadaveric dissection for the teaching of anatomy rely to a great extent on the use of 3D anatomy software such as Anatomage which is a 3D Anatomy and Virtual Dissection Platform, Complete Anatomy that is a 3D anatomy platform and other digital resources including virtual reality and augmented reality resources, e-learning modules, and patient simulations [9]. Hence, through these sessions, students have the ability to become exposed to a wide range of anatomical models that allow them to appreciate the three dimensionalities of specific anatomical structures and the anatomical relations between them. As the pandemic ceased face-to-face sessions that implies that students lost their opportunity to use these models to enhance their anatomy learning, thus, an insight into how these sessions took place amid the pandemic is necessary.

Undoubtedly, the abrupt transition from face-to-face teaching to online teaching and remote learning paved the way for technology-enhanced resources to be created or used as primary educational resources and learning tools by anatomy educators and students as well [21]. Anatomy educators often need to stretch their imagination when designing learning activities for students, irrespective of the anatomy teaching methods that are being used at the institution they work for. It is possible that during the pandemic, anatomy educators working at “cadaver free” medical schools might have created online digital anatomy educational resources including multimedia videos of their physical anatomy models, and their digital anatomy resources, to support students’ learning. The use of such non-cadaveric resources can be equally beneficial to students if designed in a way that they constructively align with specific anatomy learning objectives. Such resources can allow students to appreciate anatomical relationships between adjacent anatomical structures or isolate an anatomical structure and appreciate its three dimensionality. The documentation of such resources in the literature in the form of research articles, descriptive articles, viewpoint commentaries, or letters to the editor is currently not available or extremely limited. Hence, medical schools that rely predominantly on technology for the teaching of anatomy can provide a great insight on the resources they created or use to teach anatomy to their medical students and how these aid the students’ learning and students’ satisfaction as well.

The input of anatomy educators working at such institutions is still necessary as the pandemic is not over yet and there are still medical schools that did not return to face-to- face teaching. Furthermore, insights from anatomy educators working at such institutions can be beneficial to the wider anatomy pedagogy readership as there are more medical institutions that are now shifting away from the traditional anatomy teaching methods irrespective of whether they rely on the use of cadaveric material for the teaching of anatomy or not. Recently, a couple of medical institutions in the UK that employ cadaveric or prosection-based practicals for their anatomy teaching, needed to temporarily stop using wet donated cadaveric material due to issues related to the ventilation system in the anatomy laboratory proving the level of exposure to formaldehyde outside the suggested normal range. Hence, the use of wet donated cadaveric material is temporarily on hold meaning that only plastic physical anatomy models, digital models, and dry specimens such as plastinated specimens can be used without the need of having an effective ventilation system established in the anatomy laboratory. Insights from anatomy educators working at “cadaver-free” institutions can be beneficial to the wider anatomy pedagogy readership as there are more and more medical institutions that now employ non-cadaveric teaching-based methods for the teaching of anatomy. Input from these educators is not necessary just in terms of guiding anatomy educators who work at medical institutions that use cadavers, but it can be a starting point for collaboration between medical institutions who employ different anatomy teaching methods. A collaboration between medical schools that employ cadaveric dissection or prosection-based practicals and those that do not might be proven beneficial as anatomy educators can share their different experiences and personal insights with each other in hope to overcome common barriers that hinder the teaching of anatomy and students from learning anatomy effectively. Lastly, such collaboration can lead to the exchange of ideas in terms of creating anatomy resources that could be proven beneficial to all medical students irrespective of the anatomy teaching methods employed at the medical institution they attend.
